# Local delivery of hormonal therapy with silastic tubing for prevention and treatment of breast cancer

**DOI:** 10.1038/s41598-017-18436-1

**Published:** 2018-01-08

**Authors:** Jeenah Park, Scott Thomas, Allison Y. Zhong, Alan R. Wolfe, Gregor Krings, Manuela Terranova-Barberio, Nela Pawlowska, Leslie Z. Benet, Pamela N. Munster

**Affiliations:** 1 Division of Hematology and Oncology, University of California, San Francisco, USA; 2 Department of Molecular and Cell Biology, University of California, Berkeley, USA; 3 Department of Bioengineering and Therapeutic Sciences, University of California, San Francisco, USA; 4 Department of Pathology, University of California, San Francisco, USA

## Abstract

Broad use of germline testing has identified an increasing number of women at risk for breast cancer with a need for effective chemoprevention. We report a novel method to selectively deliver various anti-estrogens at high drug levels to the breast tissue by implanting a device comprised of silastic tubing. Optimized tubing properties allow elution of otherwise poorly bioavailable anti-estrogens, such as fulvestrant, into mammary tissue *in vitro* and *in vivo* with levels sufficient to inhibit estrogen receptor activation and tumor cell proliferation. Implantable silastic tubing delivers fulvestrant selectively to mouse mammary fat tissue for one year with anti-tumor effects similar to those achieved with systemic fulvestrant exposure. Furthermore, local delivery of fulvestrant significantly decreases cell proliferation, as assessed by Ki67 expression, most effectively in tumor sections adjacent to tubing. This approach may thereby introduce a potential paradigm shift and offer a promising alternative to systemic therapy for prevention and early interception of breast cancer.

## Introduction

Breast cancer continues to impact the lives of many women. Over 250,000 women are diagnosed with breast cancer each year and more than 40,000 will die from the disease in 2017^1^. About 5–10% of breast cancers are linked to hereditary mutations, of which those in *BRCA1* and *BRCA*2 account for the great majority of families with inherited predisposition^2^. These deleterious mutations may convey lifetime risk of breast cancer as high as 85%^3,4^. *BRCA* carriers are also at higher risk for developing secondary breast cancers after initial diagnosis in either the same or contralateral breast^5^.

In addition to *BRCA1*/*2*, mutations in *PALB2*, *ATM*, *CHEK2*, and *BRIP1* genes confer a 20–40% lifetime breast cancer risk^6^. Recommendation for risk reduction for these mutations is less clear and bilateral mastectomies are typically not recommended. Furthermore, a strong family history of breast cancer may compound the risks in known and unknown low penetrance gene mutations^7^. The affordability and increased awareness of germline testing has led to a substantial increase in women getting multigene germ line testing and now present with a definable breast cancer risk. Hence, there is a rapidly increasing number of young women with known elevated risk for breast cancer in need of prevention and early interception strategies.

Approved breast cancer prevention strategies are limited. They include risk-reducing surgery, such as bilateral mastectomy and oophorectomy, or systemic treatment with anti-estrogens such as tamoxifen. In high risk patients, bilateral mastectomy with or without accompanying oophorectomy reduces the risk of breast cancer by more than 95%^8,9^. Although effective, the considerable physical and emotional impact renders this a difficult choice for many women. A pharmacological alternative is 5 years of systemic tamoxifen treatment. To date, tamoxifen has been the only approved drug for adjuvant therapy and breast cancer prevention in premenopausal women. Despite a 50% risk reduction reported in a large randomized trial of over 13,000 patients, very few women are willing to consider tamoxifen for prevention^10,11^. The pro-estrogenic effects of tamoxifen in non-breast tissues, furthermore, present significant increased risk for endometrial cancer, and strokes are a discernible risk in older women. Raloxifene, a newer selective estrogen receptor modulator (SERM), with similar benefits to tamoxifen has also been approved for prevention but is limited to only postmenopausal women. The side effects associated with systemic exposure have similarly resulted in minimal acceptance even in women with high risk. Fulvestrant, a highly potent and active selective estrogen receptor downregulator (SERD), is currently approved for metastatic breast cancer in postmenopausal women. Despite well-established activity in postmenopausal women, its poor bioavailability has made this agent less suitable in premenopausal women and has not been used for prevention^12^.

Thus, the limited acceptable choices for breast cancer prevention strategies in an increasing number of young women emphasize a strong need for other options. Anti-estrogens delivered locally to the breast would be a promising alternative to current breast cancer prevention measures with the hope of eliminating or delaying the need for surgical interventions, such as prophylactic mastectomies, or reduce the impact from adverse side effects of systemic treatment. The goal of localized treatment is to effectively deliver the active drug to the appropriate tissue and maintain the desired therapeutic spatial distribution of the drug while minimizing systemic exposure.

Here, we investigated the potential of an implantable device comprised of silastic tubing for long-term local delivery of anti-estrogens selectively to the breast. Silastic Rx (dimethylpolysiloxane; Dow Corning Corp.) is a biomedical grade platinum-cured elastomeric silicone tubing that is routinely used in medical devices, such as shunts and medical catheters, and for drug and nutritional infusion. Unlike other polymer membranes, the silastic polymer has been shown to allow for the diffusion of various steroids^13,14^.

For this study, we tested various breast cancer drugs and metabolites to evaluate the broad application of silastic tubing implantable into mammary tissue as a depot for anti-cancer therapies selectively to the breast. We provide evidence that implantable silastic tubing can be used for long-term controlled release of fulvestrant at therapeutic concentrations sufficient to inhibit estrogen receptor signaling activation and induce apoptosis in breast cancer cells *in vitro*. Furthermore, silastic tubing delivers fulvestrant selectively to the mammary tissue and prevents cell proliferation (Ki-67) and tumor growth in mice comparable to systemic fulvestrant administration. Pharmacokinetic studies show a high drug concentration differential between the mammary tissue and other organs. Our study provides proof of concept for an implantable device comprised of silastic tubing as an effective long-term local drug delivery method for anti-estrogens selective to the breast tissue and hence an alternative for prevention and treatment of early stage breast cancer with minimal systemic exposure and toxicity.

## Results

The properties of silastic tubing suggest that steroid-based compounds can be delivered and sustained at high concentrations in its local surrounding. A device that incorporates optimized tubing diameter and wall thickness can serve as an ideal local reservoir for prolonged tissue-specific administration of an anti-cancer agent. The slow release of anti-estrogens from silastic tubing is expected to result in clinically effective local concentration within the breast parenchyma with low to minimal plasma concentrations. The high affinity of SERMs and SERDs to an estrogen-rich environment further provides a tissue advantage. We therefore sought to determine whether fulvestrant released from silastic tubing selectively to mammary tissue could prevent tumor growth.

### *In vitro* delivery of anti-estrogens to cancer cells using silastic tubing

**Figure 1:**
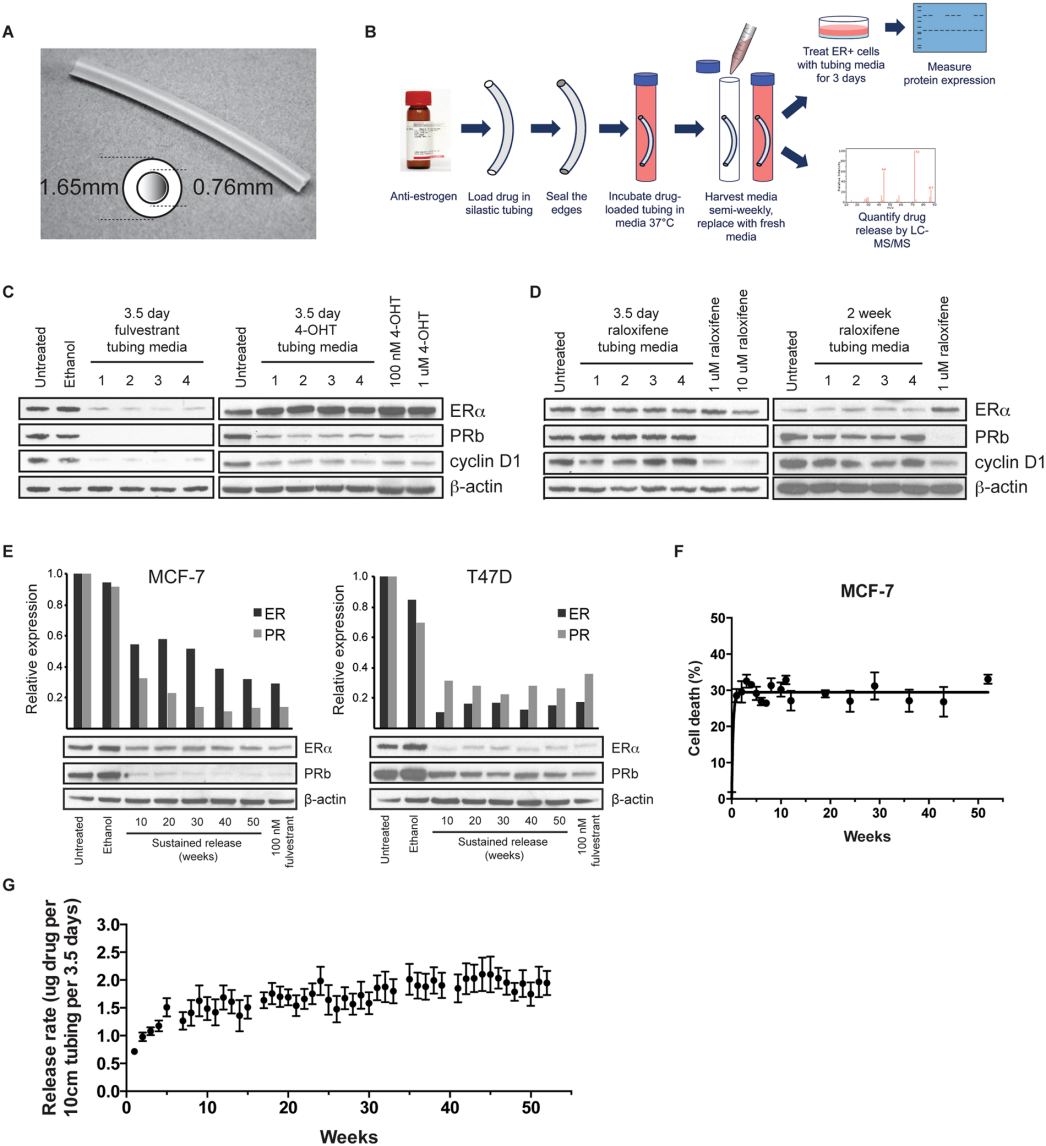
*In vitro* delivery of anti-estrogens to breast cancer cells using silastic tubing. **(A)** Silastic Rx-50 medical grade tubing with an inner diameter of 0.76 mm, an outer diameter of 1.65 mm, and a wall thickness of 0.445 mm was used for the study. **(B)** Diagram of the *in vitro* experiment to test the release of drug from silastic tubing. **(C)** Fulvestrant and 4-OHT released from silastic tubing reduced the expression of progesterone receptor (PR_b_) and cyclin D1 in MCF-7 cells. 100 nM and 1 μM 4-OHT were the concentrations of drug used to directly treat cells. Lanes 1–4 represent biological replicates. **(D)** When raloxifene-loaded tubing was incubated in media for 3.5 days or 2 weeks, it was not released at a concentration high enough to inhibit the ER signaling pathway. 1 μM and 10 μM raloxifene concentrations were used to directly treat cells. **(E)** MCF-7 and T47D cells were treated with media that was previously incubated with fulvestrant-loaded silastic tubing and collected in weeks 10, 20, 30, 40 and 50. The effect on estrogen receptor (ER) and PR_b_ expression was assessed by Western blot. β-actin was used as a loading control. **(F)** The MTS assay revealed that culturing MCF-7 cells in tubing media for 10 days causes significant cell death. **(G)** LC-MS/MS confirmed a steady release of fulvestrant in tissue culture media collected twice a week for a year (n = 4). Error bars are mean ± SEM.

We developed several device prototypes comprised of Silastic® Rx-50 Medical Grade tubing (0.76 mm inside diameter, 1.65 mm outside diameter) to test the elution characteristics of various cancer drugs and metabolites (Fig. 1A). The elution properties of fulvestrant (SERD) were compared to that of the SERMs, 4-hydroxytamoxifen (4-OHT) and raloxifene. While fulvestrant is more efficacious than other SERMs, its poor bioavailability in young women with high estrogen levels has limited its use to metastatic breast cancer in postmenopausal women^12^.

All drugs were reconstituted in ethanol at maximum solubility before they were loaded into silastic tubing of 10 cm length. After the tubing ends were sealed with Silastic® Medical Adhesive Silicone Type A (Dow Corning), devices were immersed in complete cell culture media and incubated at 37 °C. The media containing tubing was collected and replaced with fresh media twice a week (q 3.5 days). MCF-7 cells were incubated for 3 days with the collected media in which the drug was released from the tubing and compared to media in which drug was directly added (Fig. 1B).

Fulvestrant released from tubing was sufficient to downregulate estrogen receptor (ERα), progesterone receptor (PR_b_) and cyclin D1 expression (Fig. 1C). Similarly, 4-OHT transfer from tubing to media decreased PR_b_ and cyclin D1 expression, and was comparable to drug directly added to media. Media incubated with raloxifene-loaded tubing for 3.5 days, or as long as 2 weeks, did not affect ER expression or activity (Fig. 1D), suggesting that fulvestrant and 4-OHT, but not raloxifene, were released at a concentration high enough to inhibit ER signaling. Since fulvestrant does not require metabolism to an active metabolite and has no pro-estrogenic activity like tamoxifen, it was therefore chosen as the lead anti-estrogen for subsequent evaluation and optimization.

### Duration of drug delivery *in vitro* using silastic tubing

Clinical data suggests that breast cancer prevention and adjuvant therapy requires sustained delivery of anti-estrogen for 5 to 10 years for optimal benefit. To determine the rate and duration of release, fulvestrant-loaded silastic tubing was incubated in media and replaced twice weekly (q3.5d) for one year. Media from each time point was transferred to cultured MCF-7 and T47D cells for 3 days. Our data suggested that fulvestrant was released from silastic tubing for up to a year at sufficient concentration to inhibit ERα and PR_b_ expression (Fig. 1E). The observed effects were comparable to the effects seen on MCF-7 cells where fulvestrant (100 nM) was added directly to the media. Similarly, a 10-day exposure of MCF-7 cells to tubing media collected over the duration of a year caused significant cell death (Fig. 1F). Steady drug release was maintained over the course of one year without diminishing effects. Measurement of drug residuals in tubing after 52 weeks suggested that remaining drug levels would allow delivery of fulvestrant for a suggested period of 5–10 years.

In addition to determining the biological activity of fulvestrant released from silastic tubing, its concentration was quantified by liquid chromatography coupled tandem mass spectrometry (LC-MS/MS) and the release rate was calculated. Steady-state fulvestrant release was reached after about 5 weeks at a rate of 1.758 μg per 10 cm tubing per 3.5 days for the entire year (min – max: 0.829–2.794 μg) (Fig. 1G). To evaluate the effect of wall thickness and surface area on the release rate, we loaded tubing of different size with fulvestrant and replaced media twice a week (Table S1). The results revealed that the wall thickness and surface area, as determined by outside diameter, have a statistically significant effect on the amount of drug released (Table S2). More specifically, wall thickness is inversely related to the release rate whereas surface area is directly related.

### Biodistribution evaluation of locally delivered fulvestrant

**Figure 2:**
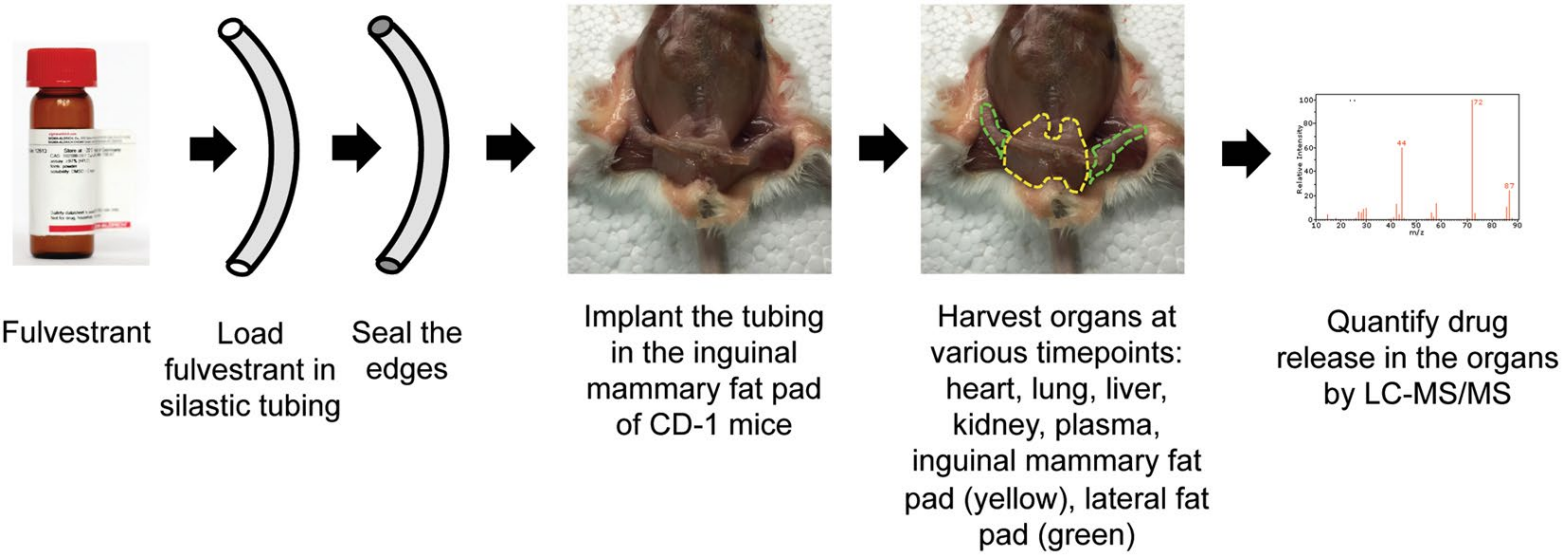
Diagram of the biodistribution experiment in CD-1 mice in which fulvestrant-loaded tubing was implanted in the mammary fat pad for various length of time.

Despite being one of the most active agents for breast cancer, fulvestrant has poor bioavailability^12^. This has limited its systemic administration in premenopausal women. The lipophilic nature of fulvestrant (log P ≈ 7.7)^15^, however, renders an ideal choice to deliver through silastic tubing and then be preferentially taken up by the adipose-rich tissue of the breast, thereby creating a high tissue-to-plasma drug differential. To determine if fulvestrant released from silastic tubing preferentially accumulates in the mammary tissue, a 2 cm piece of fulvestrant-loaded tubing was implanted in 4–6 week old female CD-1 mice proximal to the inguinal mammary fat pad (Fig. 2). Tubing length was adjusted to the width of the inguinal mammary fat pad in these mice. Drug concentrations were determined in tubing adjacent mammary fat pads (inguinal), fat pads near but not touching the tubing (abdominal), and fat pads far from the tubing (thoracic). Drug concentrations were further determined in major organs and plasma. Fulvestrant concentrations in these tissues were determined at various time points post implantation, from 1 week to 52 weeks (Table 1). Using LC-MS/MS, we determined that fulvestrant preferentially accumulates in the inguinal mammary fat pad with minimal to no detection in other organs (>300-fold difference). The concentration of fulvestrant in the inguinal mammary fat pad was >20-fold greater than those of the distant mammary fat pads (abdominal and thoracic). No gross tissue pathology was observed in the mouse during necropsy in the region surrounding the tubing or in the harvested organ (data not shown). Furthermore, body weights were measured over the 52-week study period (Figure S1) and there was no evidence of any changes in body weight patterns.

**Table 1:** LC-MS/MS revealed that there was a preferential accumulation of fulvestrant in the inguinal mammary fat pad with minimal to no detection in other organs (n = 3 or 4 mice per time point).

ng fulvestrant per g organ	Week								Average
	1	2	3	4	6	8	40	52	
IMFP	146.49 ± 52.68	208.42 ± 72.90	219.86 ± 59.10	113.26 ± 9.61	110.64 ± 17.67	140.40 ± 30.55	91.31 ± 9.96	152.87 ± 90.95	147.23 ± 16.19
AMFP	6.11 ± 0.72	5.77 ± 2.24	3.52 ± 1.05	3.37 ± 0.38	2.53 ± 0.11	3.63 ± 1.04	16.47 ± 1.48	19.29 ± 3.53	7.05 ± 1.21
TMFP	12.77 ± 7.81	5.41 ± 3.13	5.86 ± 1.66	2.28 ± 1.07	2.47 ± 0.55	2.93 ± 0.39	22.85 ± 9.72	18.17 ± 3.82	8.46 ± 1.90
heart	N.D.	0.83 ± 0.96	0.56 ± 0.56	N.D.	N.D.	N.D.	N.A.	N.A.	0.23 ± 0.17
lung	0.44 ± 0.44	0.15 ± 0.17	N.D.	N.D.	N.D.	N.D.	N.A.	N.A.	0.09 ± 0.06
liver	N.D.	N.D.	1.12 ± 1.12	5.25 ± 6.06	N.D.	N.D.	N.A.	N.A.	1.11 ± 0.96
kidney	N.D.	0.23 ± 0.26	0.02 ± 0.02	N.D.	2.33 ± 2.69	N.D.	N.A.	N.A.	0.49 ± 0.44
plasma*	1.68 ± 1.03	0.50 ± 0.19	0.68 ± 0.07	0.54 ± 0.11	0.23 ± 0.04	0.70 ± 0.29	N.A.	N.A.	0.68 ± 0.16

Abbreviations: IMFP = inguinal mammary fat pad, *plasma = ng fulvestrant/ml plasma, AMFP = abdominal mammary fat pad, N.A. = not available, TMFP = thoracic mammary fat pad, N.D. = not detectable (<0.06 ng fulvestrant per g organ).

To evaluate drug clearance at the *in vivo* level, we measured the level of fulvestrant in the fat pads six days after removing drug-loaded tubing that had been implanted for over three months. Within six days, fulvestrant concentrations in inguinal, abdominal and thoracic fat pads were below the limit of detection (n = 3 mice), suggesting the need for continued presence of drug-loaded tubing. These biodistribution experiments suggest that silastic tubing can locally deliver fulvestrant to mouse mammary tissue with minimal systemic exposure and the drug can be quickly cleared upon removal of the tubing.

### *In vivo* anti-tumor effects of systemically delivered fulvestrant

Biodistribution profiling and anti-tumor effects of systemically delivered fulvestrant were confirmed using NSG mice. The mice were randomly chosen to receive vehicle (peanut oil), 1 mg or 5 mg fulvestrant in vehicle subcutaneously once a week for five weeks. The higher weekly dose of 5 mg was chosen based on a previous study in which dramatic inhibitory effects were observed with 5 and 10 mg doses that were systemically delivered to mice once a week^16^. Fulvestrant was extracted from tissues and quantified by LC-MS/MS. The results demonstrated a lack of preferential accumulation of fulvestrant in the mammary fat pad of mice when administered via subcutaneous injection (Table 2). The weekly dose of 5 mg fulvestrant resulted in excessive drug levels in all evaluated tissues. Previous studies using a single-dose intramuscular injection of clinical fulvestrant at 250 mg in healthy female postmenopausal volunteers demonstrated that maximum plasma concentrations were approximately 11.4 ng/mL^17^. In comparison, the drug concentration detected in mouse plasma following 5 mg weekly dose was more than 100 ng/mL. At 1 mg dose per week, a significant amount of drug was found in all organs except the plasma in which it was found to be below the limit of detection. Despite the accumulation of drug in all major organs, the concentration of drug in the mammary fat pad was lower than that of the heart, lung, liver and kidney.

**Table 2:** Study of biodistribution revealed that weekly systemic delivery of fulvestrant at 5 mg dose results in excessive level of drug in every major organ. Similarly, a significant amount of drug was found in all organs but not in the plasma when mice were systemically treated with 1 mg dose per week. In contrast, the level of fulvestrant in major organs was below the limit of detection when delivered locally via silastic tubing. The IMFP could not be collected in mice that were implanted with fulvestrant-loaded tubings because it was difficult to determine where the tubings touched the fat pad once the tumors, adjoined with the tubings, were removed.

ng fulvestrant per gram of organ	Treatment		
	5 mg/week SQ injection	1 mg/week SQ injection	fulvestrant-loaded tubing
	(systemic)	(systemic)	(local)
IMFP	3,945 ± 1,134	2.93 ± 2.93	N.A.
heart	2,896 ± 2,478	152.90 ± 31.43	N.D.
lung	5,793 ± 3,651	120.80 ± 39.61	N.D.
liver	1,247 ± 929	41.95 ± 13.90	N.D.
kidney	1,389 ± 798	170.70 ± 21.31	N.D.
plasma*	128 ± 13	<3.64 ng/ml	<3.64 ng/ml

Abbreviations:*plasma = ng fulvestrant / ml plasma, IMFP = inguinal mammary fat pad, N.A. = not available, SQ = subcutaneous, N.D. = not detectable (<0.06 ng fulvestrant per g organ).

**Figure 3:**
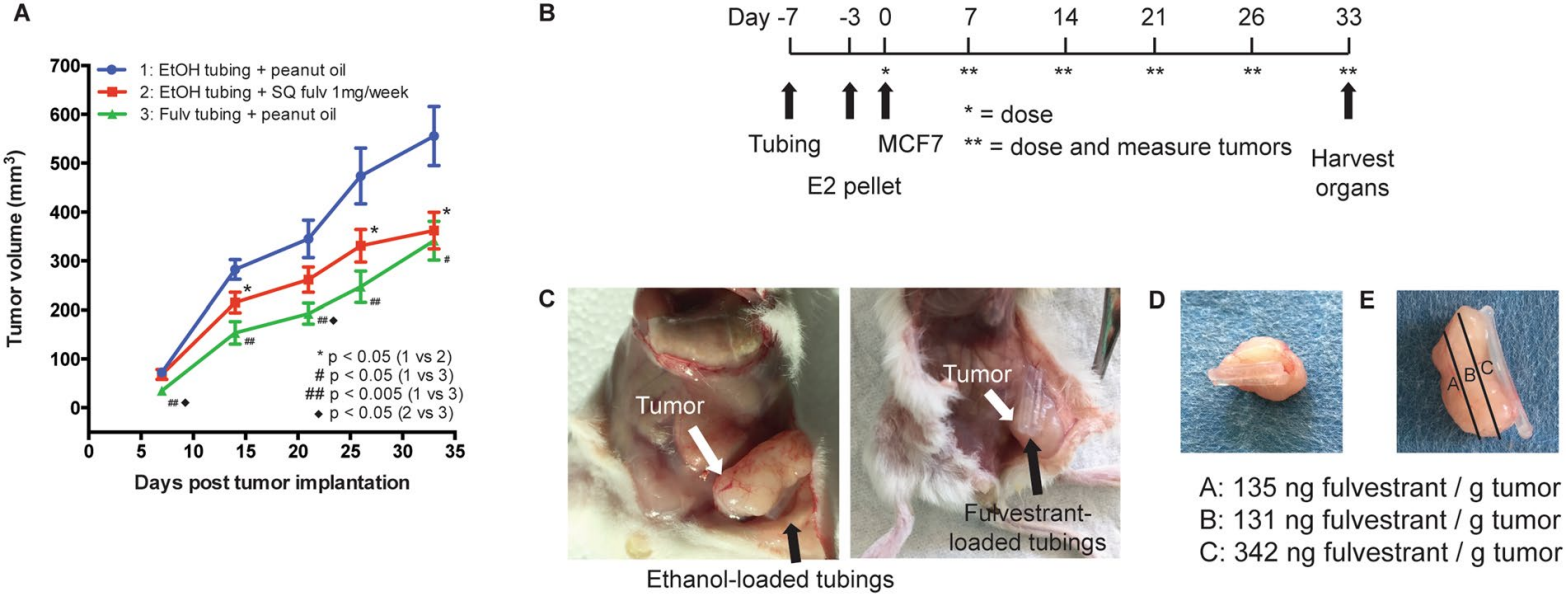
*In vivo* anti-tumor effects of systemically or locally delivered fulvestrant. **(A)** NSG mice were treated with vehicle (peanut oil), weekly subcutaneous injection of 1 mg fulvestrant or fulvestrant-loaded tubing. Drug-loaded tubing inhibited growth of MCF-7 tumors orthotopically implanted in the abdominal mammary fat pads comparable to systemically delivered fulvestrant. Error bars are mean ± SEM. **(B)** Schedule of the *in vivo* experiment to test the efficacy of local drug delivery in reducing tumor volume. **(C)** Representative images of MCF-7 xenografts treated with vehicle-loaded tubings or fulvestrant-loaded tubings in the abdominal mammary fat pads. **(D)** Representative image of MCF-7 xenograft and its adjoining tubings removed from a mouse. **(E)** LC-MS/MS confirmed the presence of fulvestrant across an entire tumor, with the highest concentration found in the section closest to the drug-loaded tubings.

Given that 5 mg weekly dosing with fulvestrant led to excessive organ drug levels in our mice, we compared the anti-tumor effect of 1 mg weekly subcutaneous dosing with fulvestrant to vehicle. This fulvestrant dose inhibited estrogen-induced tumorigenesis of MCF-7 cells orthotopically implanted in the abdominal mammary fat pads (Fig. 3A). Subcutaneous fulvestrant injections beginning on the day of tumor cell inoculation significantly reduced tumor formation, decreasing tumor volumes by 45%.

### *In vivo* anti-tumor effects of drug-loaded silastic tubing

The goal of local delivery is to enhance local drug concentrations while minimizing systemic exposure. As shown in Table 2, systemic administration of fulvestrant resulted in low mammary fat pad exposure with high systemic concentrations and considerable organ exposure. We therefore examined the efficacy of fulvestrant locally delivered by silastic tubing. We surgically placed two pieces of tubing that were either loaded with ethanol or fulvestrant on each side of the abdominal mammary fat pads. Given that implantation of drug-loaded tubing in CD-1 mice resulted in minimal to no detectable drug in the plasma, we expected that the drug released from the tubing on one side of the mouse would not affect the tumor implanted on the other side of the mouse. As such, we engrafted two tumors per mouse to preserve tumor heterogeneity (n = 8 mice per cohort). MCF-7 cells were orthotopically injected adjacent to the implanted tubing a week following tubing implantation (Fig. 3B). Tubing was introduced before the tumor cells in order to allow the drug to begin eluting and concentrating in mammary tissue to better model tumor prevention. The mice were dosed weekly with vehicle to mimic the previous experiment. Tumor growth was monitored twice a week for five weeks, and tumors and organs were harvested at the end of the study. There was no statistical difference in the body weight of mice between each cohort throughout the duration of the study (Figure S2).

Throughout the study, significant inhibition of tumor growth was observed in the group with fulvestrant-loaded tubing compared to ethanol-loaded tubing (Fig. 3A). At the end of the experiment, drug delivered by silastic tubing prevented tumor growth by 40%, akin to 1 mg/week systemic dosing. Furthermore, results from the biodistribution studies indicated that fulvestrant was undetectable in all major organs evaluated (Table 2).

Representative images of MCF-7 tumor xenografts that have grown next to ethanol-loaded tubing versus drug-loaded tubing are shown, demonstrating the difference in tumor size (Fig. 3C). MCF-7 xenograft adjoined the tubing but did not encase them (Fig. 3D). The orientation of the tumor to drug-loaded tubing was noted at the time of harvest to evaluate the effect of drug penetration through the tumor. In particular, one tumor of 5 mm in diameter harvested from a mouse implanted with drug-loaded tubing was cut into three ~1.5 mm sections parallel to the tubing: adjacent, intermediate, and distal (Fig. 3E). LC-MS/MS was performed on these sections to quantify fulvestrant penetrance. Fulvestrant was detected in all sections of tumor, with the highest concentration found in the section adjacent to the tubing (342 ng/g of tumor). The concentration of fulvestrant was comparably reduced in the intermediate (131 ng/g of tumor) and distal (135 ng/g of tumor) sections.

### Local delivery of fulvestrant reduces cell proliferation *in vivo*

**Figure 4:**
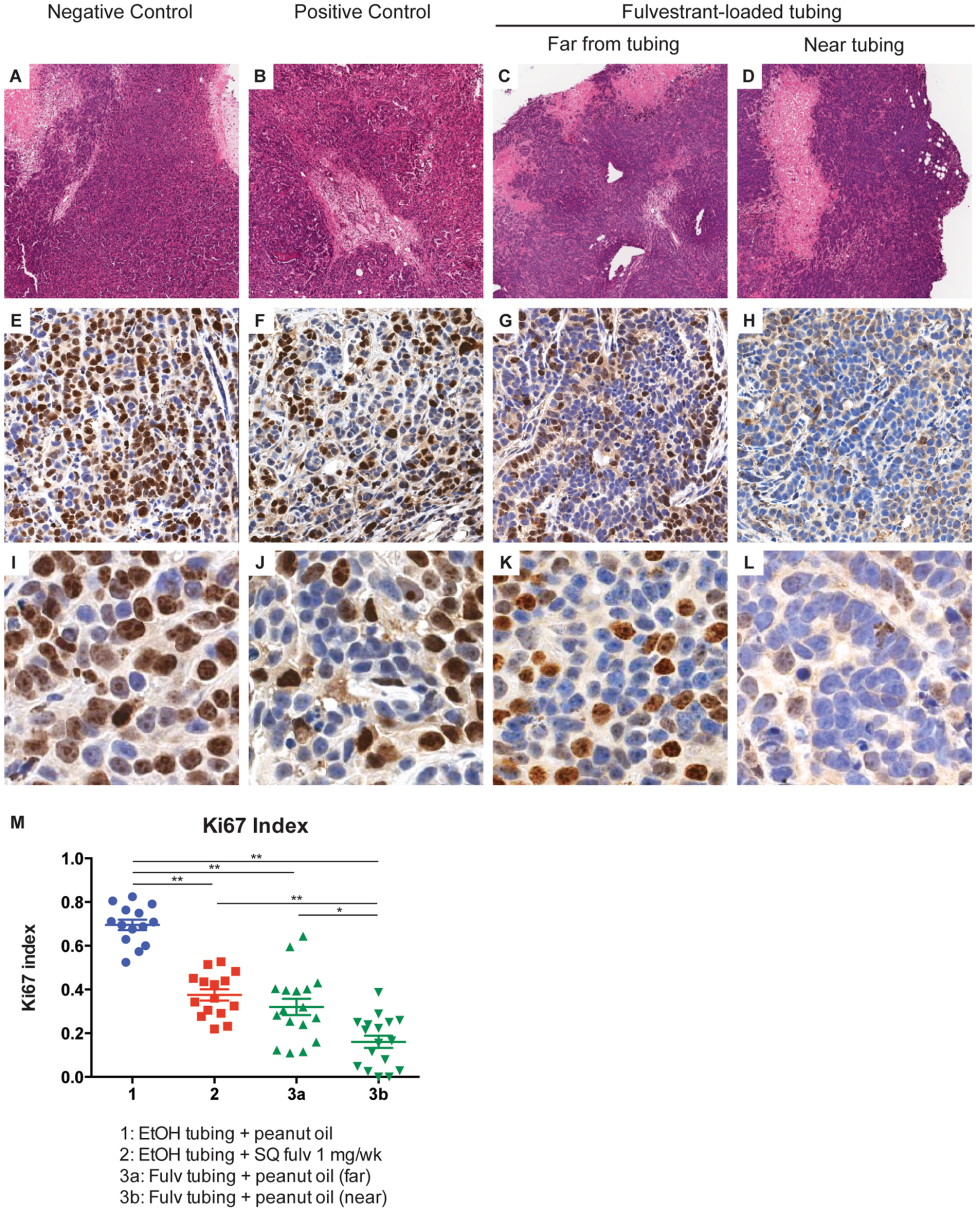
Ki67 immunohistochemical analysis of MCF-7 xenografts from NSG mice treated with local or systemic fulvestrant. Analysis was performed in tumors implanted adjacent to ethanol-loaded tubing and vehicle (negative control; A, E and I), ethanol-loaded tubing and weekly subcutaneous injection of 1 mg fulvestrant (positive control; B, F and J), or fulvestrant-loaded tubing and vehicle. For the tumors that were implanted next to fulvestrant-loaded tubings, cell proliferation in the regions of the tumor far from the tubing (**C**,**G** and **K**) was assessed separately from the region of the tumor next to the tubing (**D**,**H** and **L**). **(A–D)** Representative tissue sections of analyzed tumors (hematoxylin and eosin, 20x magnification). **(E–H)** Representative images of tumor tissue sections immunostained with Ki67, 200x magnification. **(I–L)** Representative images of tumor tissue sections immunostained with Ki67, 400x magnification. **(M)** Quantitative analysis of percentage of tumor cells with positive Ki67 staining. *p < 0.05, **p < 0.005; n = 8 mice per cohort. Error bars are mean ± SEM.

To determine the biological effects of local fulvestrant delivery, tumors were subjected to immunohistochemical staining analysis for Ki67 expression (Fig. 4A–M). There was a statistically significant decrease in Ki76 expression in tumors from fulvestrant-treated cohorts either delivered locally by silastic tubing or systemically compared to vehicle control (p < 0.005, Fig. 4M). For tumors that arose adjacent to fulvestrant-loaded tubing, Ki67 expression in sections far from the tubing (Fig. 4C,G, and K) was evaluated separately from sections near the tubing (Fig. 4D,H, and L), as a gradient of fulvestrant tumor penetrance had been previously found (Fig. 3E). There was a marked reduction in Ki67 expression in tumor regions close to fulvestrant-loaded tubing compared to more distant regions (p < 0.05; Fig. 4K–M). Tumors receiving systemically delivered fulvestrant (1 mg/week) demonstrated Ki67 expression similar to tumor sections far from fulvestrant-loaded tubing, suggesting that tubing elution is comparable to systemically delivered exposure with the exception of the very high drug concentrations seen immediately adjacent to the tubing (Fig. 4M). Collectively, these data support the hypothesis that local fulvestrant delivery reduces cell proliferation as effectively as systemic delivery and may provide a boost to an area of concern if used for local delivery for early stage breast cancer.

## Discussion

Most prevention strategies for cancer include removal of the respective organ or exposure to systemic anti-cancer therapy. Unlike other disease settings, cancer prevention is fraught with uncertainties in individual risk assessment and typically lacks early surrogate markers to predict efficacy. Hence, cancer prevention studies require very large numbers of patients, are performed in an unselected population without clearly defined risk and the benefits are often small or diluted. Thus far, very few cancer prevention strategies have been successful and even those with documented benefit, such as tamoxifen in breast cancer and finasteride for prostate cancer, have found very poor uptake in the respective at-risk population due to the undesirable systemic side effects and small magnitude of risk reduction. The opportunity to selectively treat the target organ would remove the need for surgery and circumvent systemic exposure. Local drug delivery strategies have already been successfully introduced in many non-cancer related diseases, such as cardiology.

Here, we show that silastic tubing can deliver anti-estrogens to breast tissue for a sustained period, allowing it to be used as a prevention and local therapy strategy with minimal systemic exposure. Our data demonstrate sustained and consistent release of active fulvestrant through the 52 weeks evaluated in this study. Extrapolating from the amount of residual drug left in the tubing at the end of the examined year suggests that drug release can be maintained for over 9 years. Silastic tubing released fulvestrant at therapeutic concentrations that were sufficient to inhibit ER signaling activation and tumor growth *in vitro* and *in vivo* models, with effects comparable to similar concentrations given by systemic administration of the anti-estrogen. Varying tubing sizes and wall diameters can tailor delivery to select anatomical areas, such as the outer upper quadrant of the breast that typically harbors more than 50% of tumors.

*In vivo* data suggest that silastic tubing preferentially delivers drug to mammary tissue with minimal accumulation in major organs and 20-fold lower concentrations in adjacent (abdominal) and distant fat (thoracic) pads. The ability of fulvestrant to accumulate in human fat tissue and be transferred to breast cancer cells has been recently reported^18^. These findings support the use of local drug delivery through the human breast tissue and surrounding fatty tissue. While fat pads allow for the accumulation of fulvestrant supporting the long-term use of an implanted device, the tissue rapidly cleared fulvestrant upon removal of the drug-loaded tubing. This will allow the design of devices that can be flushed to remove drug from the breast tissue and be refilled according to therapeutic needs. Consistent with fulvestrant penetrance through tumors, local delivery was more effective in reducing Ki-67 expression immediately adjacent to the tubing but maintained concentrations comparable to systemic therapy throughout the entire tumors. Our preclinical results demonstrating that silastic tubing delivery of fulvestrant can inhibit orthotopic tumor growth in a breast cancer model further support the hypothesis that this approach merits investigation with other cancer drugs.

Local drug delivery is ideally suited in a setting of local disease or recurrence with minimal risk for systemic metastases with the goal of producing high concentrations without systemic application of the drug. Our data support the use of silastic tubing as an implanted device in three major applications: early interventions for localized tumors, such as ductal carcinoma *in situ* (DCIS) or early stage breast cancer with low metastatic potential, prevention of breast cancer in women at high risk for breast cancer due genetic or hereditary predisposition, or used in concert with systemic therapy to provide a localized therapeutic boost. The current standard of care in the management of patients with DCIS is breast conservation surgery followed by adjuvant radiation therapy^19^ and systemic tamoxifen to reduce the risk for further disease in the same or contralateral breast^20–22^. Undesirable side effects and long-term sequelae, like strokes and endometrial cancer, unfortunately result in compliance rates as low as 50%^11,23^. There has been a trend for increased rates of mastectomy over lumpectomy for DCIS and low grade breast cancer in recent years even though most cases can be effectively managed with breast conserving surgery^24^. Nonetheless, perceived risk, additional screening, and the need for breast irradiation and systemic tamoxifen renders this unattractive^25^.

Fulvestrant (Faslodex®) is administered as two gluteal intramuscular injections of 250 mg each every month. Although a considerable amount is repeatedly delivered, fulvestrant elicits only mild injection site discomfort. Likewise, mice receiving localized fulvestrant for one year exhibited no gross pathology surrounding the implant. In addition, body weight of the mice steadily increased over time while relative behavior and socialization remained unchanged, together indicating the general safety of this approach.

Local delivery of drugs for breast diseases or breast cancer has been evaluated using topical adminstrations of such agents. Many of these are hormonal agents including estrogen, progestins, and active tamoxifen metabolites^26–29^. Delivered transdermally, tamoxifen may have longer retention in the local tissue^26^, but only relatively small molecules can be effectively transported through the stratum corneum in the skin^30^. A randomized study of percutaneous 4-OHT versus oral tamoxifen in women with breast cancer reported that application of gel to the skin of the breast produced consistent tumor concentrations of 4-OHT but with much lower plasma concentrations^27^. Similarly, another randomized trial showed that percutaneous administration of 4-OHT reduced tumor tissue proliferation index comparably to oral tamoxifen treatment but with a 9-fold lower systemic exposure^31^. Most recently, a randomized phase II presurgical trial concluded that the anti-proliferative effect of 4-OHT gel applied to breast skin is similar to that of oral tamoxifen in women with DCIS^29^. While topical administration of 4-OHT may eliminate first pass effects, this is not a direct local administration of the drug selectively to the breast but local absorption of a systemic drug. The feasibility and safety of direct tissue administration of progestins have been demonstrated with the use of a levonorgestrel-releasing intrauterine system approved for birth control. Mirena® provides local hormone levels at a plasma differential of > 1000 fold for up to 5 years^32^, suggesting that the proximity of the device and proclivity of the tissue for hormonal steroid uptake promote a high tissue-to-plasma differential. Our method will provide the first application of truly local drug delivery in breast cancer.

Silastic tubing may be used to deliver a variety of anti-cancer drugs to treat other types of cancers, including prostate cancer. While men with defined high risk, non-metastatic prostate cancer can benefit from prostatectomy, it carries sizable risk for impotence and incontinence^33^. Adjuvant androgen deprivation therapy (ADT) has detrimental effects on the quality of life and increases the risk for diabetes, cardiovascular disease and cognitive dysfunction^34,35^. For men with high risk for recurrence who are unwilling or unable to undergo prostatectomy or ADT, locally implanting drug-loaded silastic tubing may be as a feasible alternative to current treatments. Local androgen deprivation via silastic tubing could reduce the burden of systemic hormonal therapy while preserving the benefits of ADT. Moverover, an increasing focus on immunotherapy and DNA repair inhibition agents has provided great promise for patients with cancer^36,37^. Immunotherapy and DNA repair agents can result in rare but considerable systemic side effects, such as multi-organ inflammation from immunotherapy^38^ and acute leukemia from PARP inhibitors^39^. Such serious adverse events could potentially be resolved by a local adminstration of the agents.

There are several considerations that must be further investigated in order to maximize the benefits of local drug therapy. Efficacy of local delivery depends on the accessibility of the delivered drug to the tumor and the adjacent tissue. Thus, a thorough understanding of how a drug penetrates at the site of disease in a large animal model and then in women will be the next steps. The process is most likely dependent upon both tissue composition as well as drug characteristics. Additionally, drug formulation as well as the configuration of silastic tubing may play an integral role in altering the rate of release. Lastly, characterizing the healing and inflammatory response caused by the placement of silastic tubing and the accumulation of the drug at the site of implantation would advance this approach towards clinical practice. Further steps including large animal studies will be needed to better understand pharmacokinetic and pharmacodynamic parameters and to test device protoypes in a more size-appropriate environment. To this end, female alpine goats will most likely be used due to the udders’ similarity in size and physiology to the human breast. Using an alpine goat model may be a tool to understand the diffusion of drug through mammary tissue over time, the biodistribution of released drug in major organs, fibrotic capsule formation in proximity to the implant, as well as resultant organ pathology and toxicity prior to clinical testing. The long standing safety data on silastic tubing, on the other hand, will allow rapid clinical translation. In summary, a localized and sustained delivery of approved anti-cancer agents could allow novel approaches to cancer prevention and local therapy in breast and other tissues.

## Methods

### Materials

Dulbecco’s Modified Eagle Medium (DMEM) was purchased from Mediatech (Tewksbury, MA). Fetal bovine serum (FBS) was purchased from GE Healthcare (Logan, UT). Penicillin-streptomycin was purchased from UCSF Cell Culture Facility (San Francisco, CA). Fulvestrant was purchased from Sigma (St. Louis, MO). Raloxifene was purchased from Santa Cruz Biotechnology (Dallas, TX). 4-hydroxytamoxifen (4-OHT) was purchased from Calbiochem (San Diego, CA). Antibody against β-actin was purchased from Sigma (St. Louis, MO). Antibodies against ERα and cyclin D1 were purchased from Santa Cruz Biotechnology (Dallas, TX). 3-mm pellets containing 0.36 mg of 17β-estradiol was purchased from Innovative Research of America (Sarasota, FL).

Silastic Rx-50 medical grade tubing and silastic medical adhesive silicone type A were purchased from Dow Corning (Auburn, MI). We used silastic Rx-50 tubing that has an inner diameter of 0.76 mm and an outer diameter of 1.65 mm with a wall thickness of 0.445 mm. The ends of the tubing were sealed with Silastic® Medical Adhesive Silicone Type A (Dow Corning). This adhesive contains no solvent and cures at room temperature upon exposure to atmospheric moisture. Once fully cured, the resulting silicone elastomer is known to have the general composition of conventional silicone elastomer.

### Cell Culture

MCF-7 and T47D cells were obtained from the American Type Culture Collection (Manassas, VA). Cell lines used in this study were authenticated by short tandem repeat (STR) profiling on Promega PowerPlex16HS Assay at University of Arizona Genetics Core (Tucson, AZ). Cells were grown in DMEM supplemented with 10% FBS and 1% penicillin-streptomycin. They were maintained in a humidified incubator with 5% CO_2_ atmosphere at 37 °C.

### Silastic tubing preparation for *in vitro* release analysis

After silastic tubing was cut in the appropriate length, it was loaded with fulvestrant that was reconstituted at maximum soluble concentration in 100% ethanol (27.5 mM). The ends of the tubing were sealed with silastic medical adhesive silicone type A and cured for 3 days at room temperature. The tubing was incubated in 5 ml of DMEM supplemented with 10% FBS and 1% penicillin-streptomycin on a rocker at 37 °C. Every 3.5 days, old media was collected and fresh media was added. The media samples were kept at −20 °C until ready to use. MCF-7 and T47D cells were treated with the tubing media for 3 days, unless otherwise stated.

### Western Blot

Cells were lysed in RIPA buffer (Sigma) containing 1% Halt phosphatase and protease inhibitor cocktail (Thermo). Total protein was estimated using Pierce BCA Protein Assay Kit (Thermo). Samples were denatured at 100 °C for 5 min. Equal amounts of proteins were loaded on Bolt Bis-Tris Plus 4–12% gel (Invitrogen) and transferred to PVDF membranes (Millipore). The membranes were blocked with 5% non-fat milk for 1 hour and incubated overnight at 4 °C with primary antibodies diluted accordingly in 0.1% TBST: PR_b_ at 1:1000, ERα at 1:5000, cyclin D1 at 1:2500, and β-actin at 1:20000. They were washed 6 times for 5 min each with 0.1% TBST and incubated for 1 hour at room temperature with appropriate HRP-conjugated secondary antibodies diluted 1:20000. After washing the membrane 6 times for 5 min with 0.1% TBST, the signal was detected by Amersham ECL Prime Western Blotting Detection Reagents (GE Healthcare). Quantitative comparisons between samples have been done on the same blot. ImageJ software was used to quantify the bands.

### Cell viability assay

To assess viability of MCF-7 cells, an MTS assay was performed using CellTiter 96 AQueous one solution (Promega). A 96-well tissue culture plate was seeded with 2000 cells per well and allowed to adhere for two nights. Cells were then treated with 200 μl media (100 μl of tubing media diluted in 100 μl fresh complete media) for 10 days. The media was then removed and cells were incubated with 100 μl assay reagent (1:5, CellTiter reagent to PBS (v/v)) at 37 °C for 2–3 hours. Absorbance at 492 nm was measured as readout for cellular activity.

### LC-MS/MS

Fulvestrant concentrations were determined by a liquid chromatography-tandem mass spectrometry (LC-MS/MS) method using fulvestrant-d_3_ as the internal standard. To quantify the level of fulvestrant in tissue culture media and mouse plasma, frozen samples were thawed at ambient temperature and 100 μl of each sample was used for analysis. The samples were purified by protein precipitation using 200 μl 100% methanol/0.1% formic acid. They were incubated at −20 °C for 30 min and then centrifuged at 16,000xg for 10 min. The supernatants were transferred to a vial and 10 μl aliquot was analyzed. Liquid chromatography was carried out with a Shimadzu Prominence HPLC and a 4.6 × 50 mm, 5 μm, 100 Å Kinetex core-shell C18 column (Phenomenex). An isocratic mobile phase (94% methanol:water, 0.1% acetonitrile, 0.1% formic acid, 160 mg/L ammonium acetate) was pumped at 0.5 mL/min. The run time was 3 min and analyte retention was 1.43 min, with a diverter valve only open to the MS/MS from 1.3 to 2.0 min. Detection was performed with a Sciex API-4000 mass spectrometer with electrospray ionization in the positive ion mode operated by Analyst 1.6 software (AB Sciex). The transitions used were m/z 607.6 → 467.2 and 610.6 → 468.5 for the analyte and internal standard, respectively. The MS/MS was set to 37 and 39 eV for EC (collision energy), respectively. Settings in common for both molecules were DP (declustering potential) = 81 V, CXP (collision cell exit potential) = 22 V, EP (entrance potential) = 10.5 V, IS (ion spray voltage) = 5,500 V, temperature = 600 °C, CAD (collision gas) = 12 lbf in^−2^, CUR (curtain gas) = 35 lbf in^−2^ and GS1 (ion source nebulizer gas) = GS2 (ion source heater gas) = 50 lbf in^−2^.

The fulvestrant standard curve went from 4 to 600 nM and samples were spiked during the protein precipitation step with 10 μl of 10 μM deuterated fulvestrant in Analyst software. Fulvestrant peak areas were normalized to the internal standard and plotted against concentration (r > 0.99). The limit of detection for fulvestrant in tissue culture media and mouse plasma was 4–6 nM. The intraday and interday precisions were within 3% and 10%, respectively.

To assess the level of fulvestrant in mouse tissues from the biodistribution study, samples (100–300 mg) were homogenized in 450 μl PBS using a TissueLyser (Qiagen) for 5 min. As an internal control, 50 μl of 1 μM deuterated fulvestrant was added to each sample. Then 1 ml 80% methanol/20% 0.2 M ZnSO_4_ protein precipitant was added to the tissue homogenates and immediately vortexed. Samples were incubated at −20 °C for 30 min and then centrifuged at 16,000 × g for 10 minutes. The supernatant was diluted 1:2 in distilled water and loaded onto a 3 ml Oasis HLB cartridge (Waters) that had been pre-conditioned with 2.5 ml methanol followed by 2.5 ml 20% methanol/water. The cartridge was rinsed with 2.5 ml 60% methanol/1 mM NaOH, 2.5 ml 70% methanol/0.01 M HCl, followed by 2.5 ml 80% methanol, followed by 150 μl of 90% methanol. Fulvestrant was eluted with 2 ml of 90% methanol. The solvent was evaporated using a Speedvac and fulvestrant was reconstituted with 100 μl of mobile phase. Reconstituted fulvestrant was transferred to a vial and 10 μl aliquot was analyzed by LC-MS/MS. The column, flow setting, instrumentation and MS/MS settings were as above. However, the LC method was altered to better wash the column after each sample, as follows: 0.1 to 1.8 min, 94% methanol:water; 1.8 to 2.1 min, linear ramp to 100% methanol; 2.1 to 2.6 min, 100% methanol; 2.6 to 2.75 min, linear ramp to 94% methanol; 2.75 to 3.7 min, 94% methanol. The same mobile phase additives as above were continually present, and retention and diverter valve settings were unchanged. Fulvestrant peak areas were normalized to the internal standard and plotted against concentration (r > 0.98). The limit of detection for fulvestrant in mouse tissues was 20–40 nM.

### *In vivo* mouse studies

For the biodistribution study, 4–6 week old female CD-1 mice were obtained from the UCSF Breeding Core. After fulvestrant was reconstituted in 100% ethanol, it was loaded in 2 cm piece of medical grade silastic tubing. Once the ends of the tubing were sealed, fulvestrant-loaded silastic tubing was surgically implanted proximal to the inguinal mammary fat pad. At each time point post-implantation, blood samples from 3–4 anaesthetized mice were collected in heparinized tubes via cardiac puncture and plasma was separated by centrifugation. In addition, various organs were harvested and stored in −80 °C until required for analysis. Fulvestrant was extracted from plasma and tissues with a protein precipitation method and analyzed by LC-MS/MS.

For the efficacy study, 4–5 week old female NOD.Cg-*Prkdc* ^*scid*^ *Il2rg* ^*tm1Wjl*^/SzJ (NSG) mice were obtained from the UCSF Breeding Core. Two pieces of 1.6 cm tubing that were loaded with either ethanol or fulvestrant within the 1 cm center lumen were implanted on each side of the abdominal mammary fat pads. Tubing length was chosen based on the size of NSG mice. Four days afterwards, a 60-day release 0.36 mg estradiol pellet was implanted subcutaneously in the dorsal posterior region. Three days later, exponentially growing MCF-7 cells were orthotopically implanted in the left and right mammary fat pad (5 × 10^6^ cells per graft) to mimic the microenvironment of breast cancer. MCF-7 cells were prepared by the addition of trypsin-EDTA, washed with complete media, collected, and resuspended in 50% DMEM and 50% Matrigel. For systemic delivery, mice were subcutaneously injected with 1 mg or 5 mg of fulvestrant suspended in vehicle (peanut oil) every week for five weeks starting from the day of tumor cell implantation. For local delivery, mice were injected with vehicle only every week for five weeks starting from the day of tumor cell implantation. Tumor growth (width^2^ × length/2) and body weight were monitored twice a week with a digital caliper. At the end of the treatment period or when tumor volume exceeded 1,000 mm^3^, tumors and tissue samples were harvested for analysis. Animal studies were conducted according to a UCSF Laboratory Animal Resource Center approved protocol (AN090303).

### Histology and immunohistochemistry

After the tissues were fixed in 4% formalin for 24 hours and embedded in paraffin, 5 μm sections were cut and mounted on plus glass slides. Slides were stained with hematoxylin and eosin (H&E) by the UCSF Helen Diller Family Comprehensive Cancer Center Tissue Core Laboratory for morphologic analysis of the tumors. The proliferative index of MCF-7 tumor xenografts was determined by immunohistochemical detection of Ki67 expression. Specifically, sections were deparaffinized in xylene and then hydrated through exposure with graded alcohols (100%, 95%, 70%, 50%, water). The slides were immersed in antigen retrieval solution (Vector Lab) and heated for 10 min. After cooling to room temperature, the slides were permeabilized in TBST for 15 min, rinsed in water, and subsequently incubated with 3% hydrogen peroxide for 10 min to quench endogenous peroxidase activity (Sigma). To block non-specific binding, the slides were incubated with blocking solution (Vector Lab) for 30 min. They were incubated with Ki67 primary antibodies (Invitrogen 180191Z, 1:100) overnight at 4 °C. As negative controls, the primary antibodies were replaced with blocking solution. After washing the primary antibodies with TBST, peroxidase-labeled polymer conjugated to goat anti-rabbit immunoglobulins (Santa Cruz) were added for 30 min. For signal detection, the slides were incubated with Elite ABC-chromogen Reagent (Vectastain) for 30 min and diaminobenzidine mixture (Vector Lab) for up to 5 min. Slides were counterstained with hematoxylin (Sigma), dehydrated in graded alcohols, and subsequently mounted in mounting media (Dako). Whole slide images of H&E and Ki67 immunostained tumor sections were scanned using an Aperio ScanScope XT whole slide scanner (Aperio) and visualized using ImageScope software (Leica). Ki67 proliferation index was scored and quantitated as the average percentage of Ki67+ tumor cell nuclei per total tumor cell nuclei in 2–3 captured fields per sample using Immunoratio software. At least 200 cells were counted per field.

### Statistical analysis

Data are expressed as averages with the standard error of the mean (±SEM) indicated. Graphs were created with Prism software. Two-sided non-paired Student’s *t* test was used to determine differences between two groups, with p < 0.05 considered statistically significant.

### Data availability statement

The datasets supporting the conclusions of this study are included within this published article and its supplementary files.

### Ethics approval

All animal procedures were approved by the UCSF Animal Care and Use Committee.

## Electronic supplementary material

Supplemental Information

## References

[1] Siegel RL, Miller KD, Jemal A (2017). Cancer Statistics, 2017. CA Cancer J Clin.

[2] Campeau PM, Foulkes WD, Tischkowitz MD (2008). Hereditary breast cancer: new genetic developments, new therapeutic avenues. Hum Genet.

[3] Antoniou A (2003). Average risks of breast and ovarian cancer associated with BRCA1 or BRCA2 mutations detected in case Series unselected for family history: a combined analysis of 22 studies. Am J Hum Genet.

[4] Chen S, Parmigiani G (2007). Meta-analysis of BRCA1 and BRCA2 penetrance. J Clin Oncol.

[5] Metcalfe K (2014). Contralateral mastectomy and survival after breast cancer in carriers of BRCA1 and BRCA2 mutations: retrospective analysis. BMJ.

[6] Shiovitz S, Korde LA (2015). Genetics of breast cancer: a topic in evolution. Ann Oncol.

[7] Pharoah PD, Day NE, Duffy S, Easton DF, Ponder BA (1997). Family history and the risk of breast cancer: a systematic review and meta-analysis. Int J Cancer.

[8] Domchek SM (2010). Association of risk-reducing surgery in BRCA1 or BRCA2 mutation carriers with cancer risk and mortality. JAMA.

[9] Kauff ND (2008). Risk-reducing salpingo-oophorectomy for the prevention of BRCA1- and BRCA2-associated breast and gynecologic cancer: a multicenter, prospective study. J Clin Oncol.

[10] Fisher B (2005). Tamoxifen for the prevention of breast cancer: current status of the National Surgical Adjuvant Breast and Bowel Project P-1 study. J Natl Cancer Inst.

[11] van Herk-Sukel MP (2010). Half of breast cancer patients discontinue tamoxifen and any endocrine treatment before the end of the recommended treatment period of 5 years: a population-based analysis. Breast Cancer Res Treat.

[12] Robertson JF (2004). Selective oestrogen receptor modulators/new antioestrogens: a clinical perspective. Cancer Treat Rev.

[13] Nash HA, Robertson DN, Moo Young AJ, Atkinson LE (1978). Steroid release from silastic capsules and rods. Contraception.

[14] Kumar D, Farooq A, Laumas KR (1981). Fluid-filled silastic capsules: a new approach to a more constant steroidal drug delivery system. Contraception.

[15] AstraZeneca, Environmental Risk Assessment Data: https://www.astrazeneca.com/content/dam/az/our-company/sustainability/fulvestrant.pdf. (2015).

[16] Osborne CK (1995). Comparison of the effects of a pure steroidal antiestrogen with those of tamoxifen in a model of human breast cancer. J Natl Cancer Inst.

[17] Robertson JF, Harrison M (2004). Fulvestrant: pharmacokinetics and pharmacology. Br J Cancer.

[18] Thomas S (2017). Autologous Fat Grafting as a Novel Antiestrogen Vehicle for the Treatment of Breast Cancer. Plast Reconstr Surg.

[19] Shah C (2015). Ductal Carcinoma *In Situ* of the Breast: Evaluating the Role of Radiation Therapy in the Management and Attempts to Identify Low-risk Patients. Am J Clin Oncol.

[20] Fisher B (1999). Tamoxifen in treatment of intraductal breast cancer: National Surgical Adjuvant Breast and Bowel Project B-24 randomised controlled trial. Lancet.

[21] Worni M (2015). Trends in Treatment Patterns and Outcomes for Ductal Carcinoma *In Situ*. J Natl Cancer Inst.

[22] Joslyn SA (2006). Ductal carcinoma *in situ*: trends in geographic, temporal, and demographic patterns of care and survival. Breast J.

[23] Hadji P (2013). Persistence in patients with breast cancer treated with tamoxifen or aromatase inhibitors: a retrospective database analysis. Breast Cancer Res Treat.

[24] Hwang ES (2010). The impact of surgery on ductal carcinoma *in situ* outcomes: the use of mastectomy. J Natl Cancer Inst Monogr.

[25] Martinez KA, Fagerlin A, Witteman HO, Holmberg C, Hawley ST (2016). What Matters to Women When Making Decisions About Breast Cancer Chemoprevention?. Patient.

[26] Mauvais-Javis P, Baudot N, Castaigne D, Banzet P, Kuttenn F (1986). trans-4-Hydroxytamoxifen concentration and metabolism after local percutaneous administration to human breast. Cancer Res.

[27] Pujol H (1995). Phase I study of percutaneous 4-hydroxy-tamoxifen with analyses of 4-hydroxy-tamoxifen concentrations in breast cancer and normal breast tissue. Cancer Chemother Pharmacol.

[28] Sitruk-Ware, R., Seradour, B., Lafaye, C. in *Percutaneous Absorption of Steroids*., P. Jarvis, C. F. Vickers, J. Wepierre, Eds., pp. 219–229 (Academic Press, London, 1980).

[29] Lee O (2014). A randomized phase II presurgical trial of transdermal 4-hydroxytamoxifen gel versus oral tamoxifen in women with ductal carcinoma *in situ* of the breast. Clin Cancer Res.

[30] Prausnitz MR, Langer R (2008). Transdermal drug delivery. Nature biotechnology.

[31] Rouanet P (2005). Neoadjuvant percutaneous 4-hydroxytamoxifen decreases breast tumoral cell proliferation: a prospective controlled randomized study comparing three doses of 4-hydroxytamoxifen gel to oral tamoxifen. J Clin Oncol.

[32] Nilsson CG, Haukkamaa M, Vierola H, Luukkainen T (1982). Tissue concentrations of levonorgestrel in women using a levonorgestrel-releasing IUD. Clin Endocrinol (Oxf).

[33] Roth AJ, Weinberger MI, Nelson CJ (2008). Prostate cancer: psychosocial implications and management. Future Oncol.

[34] D’Amico AV (2007). Influence of androgen suppression therapy for prostate cancer on the frequency and timing of fatal myocardial infarctions. J Clin Oncol.

[35] Keating NL, O’Malley AJ, Smith MR (2006). Diabetes and cardiovascular disease during androgen deprivation therapy for prostate cancer. J Clin Oncol.

[36] Apetoh L, Ladoire S, Coukos G, Ghiringhelli F (2015). Combining immunotherapy and anticancer agents: the right path to achieve cancer cure?. Ann Oncol.

[37] Lord CJ, Tutt AN, Ashworth A (2015). Synthetic lethality and cancer therapy: lessons learned from the development of PARP inhibitors. Annu Rev Med.

[38] Weber JS, Yang JC, Atkins MB, Disis ML (2015). Toxicities of Immunotherapy for the Practitioner. J Clin Oncol.

[39] Kim G (2015). FDA Approval Summary: Olaparib Monotherapy in Patients with Deleterious Germline BRCA-Mutated Advanced Ovarian Cancer Treated with Three or More Lines of Chemotherapy. Clin Cancer Res.

