# Supplementary material for: Local delivery of hormonal therapy with silastic tubing for prevention and treatment of breast cancer
Source: Sci Rep. 2018 Jan 8;8:92. doi: 10.1038/s41598-017-18436-1 (PMC5758798; doi:10.1038/s41598-017-18436-1)
Supplement: Supplementary file 1 — Supplemental Information [file 41598_2017_18436_MOESM1_ESM.pdf]

Title: Local delivery of hormonal therapy with silastic tubing for prevention and treatment of breast cancer

Authors: Jeenah Park[1], Scott Thomas[1], Allison Y. Zhong[2], Alan R. Wolfe[3], Gregor Krings[4], Manuela Terranova-Barberio[1], Nela Pawlowska[1], Leslie Z. Benet[3], Pamela N. Munster[1*]

Affiliations:
[1] Division of Hematology and Oncology, University of California, San Francisco
[2] Department of Molecular and Cell Biology, University of California, Berkeley
[3] Department of Bioengineering and Therapeutic Sciences, University of California, San Francisco
[4] Division of Pathology, University of California, San Francisco
* Corresponding author

SUPPLEMENTAL MATERIALS

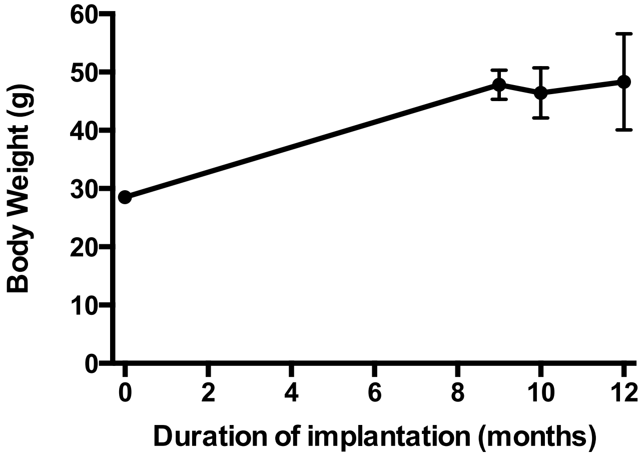

Figure S1. Body weight of CD-1 mice in which fulvestrant-loaded tubing was implanted proximal to the inguinal mammary fat pad. Error bars are mean ± SEM.

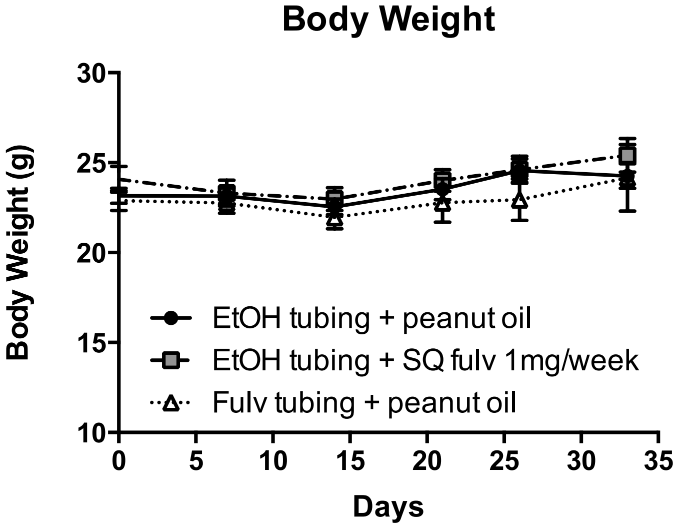

Figure S2. Body weight of NSG mice treated with locally or systemically delivered fulvestrant.

Size	Wall thickness (mm)	Inside diameter (mm)	Outside diameter (mm)
1	0.215	0.51	0.94
2	0.275	0.64	1.19
3	0.445	0.76	1.65
4	0.245	1.47	1.96
5	0.42	1.57	2.41
6	0.805	1.57	3.18
7	0.6	1.98	3.18

Table S1. Various sizes of Silastic Rx-50 Medical Grade Tubing.

	B Coefficient ± SE	95% CI	p-value
Wall thickness (mm)	-1702 ± 500	(-2748 to -656)	0.003
Outside diameter (mm)	381 ± 124	(120 to 641)	0.006

Table S2. Multiple linear regression analysis for factors determining fulvestrant release from silastic tubing.
